# Systemic Immune Modulation Alters Local Bone Regeneration in a Delayed Treatment Composite Model of Non-Union Extremity Trauma

**DOI:** 10.3389/fsurg.2022.934773

**Published:** 2022-07-07

**Authors:** Casey E. Vantucci, Tyler Guyer, Kelly Leguineche, Paramita Chatterjee, Angela Lin, Kylie E. Nash, Molly Ann Hastings, Travis Fulton, Clinton T. Smith, Drishti Maniar, David A. Frey Rubio, Kaya Peterson, Julia Andraca Harrer, Nick J. Willett, Krishnendu Roy, Robert E. Guldberg

**Affiliations:** ^1^ Wallace H. Coulter Department of Biomedical Engineering, Georgia Institute of Technology and Emory University, Atlanta, GA, United States of America; ^2^ Parker H. Petit Institute for Bioengineering and Bioscience, Georgia Institute of Technology, Atlanta, GA, United States of America; ^3^ Knight Campus or Accelerating Scientific Impact, University of Oregon, Eugene, OR, United States of America; ^4^ Marcus Center for Therapeutic Cell Characterization and Manufacturing, Georgia Institute of Technology, Atlanta, GA, United States of America; ^5^ The Atlanta Veterans Affairs Medical Center Atlanta, Decatur, GA, United States of America; ^6^ Department of Orthopaedics, Emory University, Atlanta, GA, United States of America

**Keywords:** immune dysregulation, non-union, musculoskeletal trauma, MDSCs, S100A8/A9

## Abstract

Bone non-unions resulting from severe traumatic injuries pose significant clinical challenges, and the biological factors that drive progression towards and healing from these injuries are still not well understood. Recently, a dysregulated systemic immune response following musculoskeletal trauma has been identified as a contributing factor for poor outcomes and complications such as infections. In particular, myeloid-derived suppressor cells (MDSCs), immunosuppressive myeloid-lineage cells that expand in response to traumatic injury, have been highlighted as a potential therapeutic target to restore systemic immune homeostasis and ultimately improve functional bone regeneration. Previously, we have developed a novel immunomodulatory therapeutic strategy to deplete MDSCs using Janus gold nanoparticles that mimic the structure and function of antibodies. Here, in a preclinical delayed treatment composite injury model of bone and muscle trauma, we investigate the effects of these nanoparticles on circulating MDSCs, systemic immune profiles, and functional bone regeneration. Unexpectedly, treatment with the nanoparticles resulted in depletion of the high side scatter subset of MDSCs and an increase in the low side scatter subset of MDSCs, resulting in an overall increase in total MDSCs. This overall increase correlated with a decrease in bone volume (*P* = 0.057) at 6 weeks post-treatment and a significant decrease in mechanical strength at 12 weeks post-treatment compared to untreated rats. Furthermore, MDSCs correlated negatively with endpoint bone healing at multiple timepoints. Single cell RNA sequencing of circulating immune cells revealed differing gene expression of the SNAb target molecule S100A8/A9 in MDSC sub-populations, highlighting a potential need for more targeted approaches to MDSC immunomodulatory treatment following trauma. These results provide further insights on the role of systemic immune dysregulation for severe trauma outcomes in the case of non-unions and composite injuries and suggest the need for additional studies on targeted immunomodulatory interventions to enhance healing.

## Introduction

Despite the significant clinical challenge posed by fracture non-unions, the mechanisms and biology underlying non-unions are still poorly understood. Non-unions occur in approximately 1.9%–4.9% of fractures worldwide; however, the presence of concomitant soft tissue damage or infection drastically increases this risk (1, 2). Because of this, patients with composite injuries often face multiple interventions, extended rehabilitation time, increased treatment costs, and even amputation or significant long-term disability (3–5). Improved treatment strategies with foundations in the biology and mechanisms of non-union will be essential to improve patient outcomes, especially in challenging bone healing scenarios like composite tissue injuries.

The advent of osteoimmunology resulted in an increased focus on the local relationship between the immune system and functional bone regeneration (6). More recently, the role of the *systemic* immune response has been highlighted as a contributing factor to poor outcomes and increased susceptibility to complications, such as infections, following severe musculoskeletal injury (7, 8). Traumatic injury, especially for open fractures, results in a large increase in damage-associated molecular patterns (DAMPs) and pathogen-associated molecular patterns (PAMPs) (9). This subsequently sparks a large-scale systemic inflammatory response, often termed the systemic inflammatory response syndrome (SIRS), resulting in an increase of inflammatory cells and mediators (ex: T cells, IL-1, IL-6, TNFa). Left unchecked, this response can result in tissue damage, leading to multiple organ failure or even death. To protect against this, a concurrent, systemic anti-inflammatory response, termed the compensatory anti-inflammatory response syndrome (CARS), results in upregulation of anti-inflammatory cells and mediators (ex: MDSCs, Tregs, IL-10, TGFb). In patients with uncomplicated outcomes, these two systemic responses balance each other out and systemic immune homeostasis is restored (10, 11). However, in patients who experience complications, such as non-unions, the CARS response often overwhelms the SIRS response, leading to systemic immune dysregulation and immunosuppression, marked by mediators such as myeloid-derived suppressor cells (MDSCs) and interleukin-10 (IL-10) (12, 13).

MDSCs are a heterogeneous and immature population of immunosuppressive myeloid-lineage cells that dramatically expand in response to traumatic injury during emergency myelopoiesis (12, 14). MDSCs potently suppress immune function, in particular T cells, through factors such as arginase, IL-10, TGFb, and reactive oxygen and nitrogen species (ROS/RNS) (14, 15). MDSCs have also been shown to promote expansion and activity of regulatory T cells, another immunosuppressive cell population, creating a feedforward mechanism to maintain immunosuppression (16). Recent work in multiple rat trauma models has demonstrated a relationship between systemic levels of MDSCs and bone healing outcomes. For example, in a rat non-union model, elevated blood levels of MDSCs and IL-10 negatively correlated with endpoint bone regeneration at multiple timepoints, including as early as 1-week post-treatment (17, 18). These data were further supported in another study using the same rat non-union model and a rat composite trauma model in which circulating blood MDSCs negatively correlated with defect bone volumes and the rats with the highest defect bone volumes had significantly lower MDSCs compared to rats with the lowest defect bone volumes (19). Lastly, in a rat model of infected trauma, infected rats exhibited significantly higher circulating MDSCs, significantly decreased circulating T cells, and a dysregulated cytokine response compared to uncomplicated non-infected fracture (20). These studies together suggest that systemic immune dysregulation and immunosuppression play an important role in uncomplicated healing outcomes and that MDSCs could be a therapeutic target to restore systemic immune homeostasis, create a permissive and pro-regenerative immune environment, and ultimately improve bone healing outcomes in conjunction with a local treatment strategy.

The Roy lab has previously fabricated bifunctional Janus gold nanoparticles that mimic the structure and function of antibodies, containing an Fc-mimicking peptide on one half of the particle surface and an MDSC-targeting peptide on the other half of the particle surface. These particles were used in a mouse tumor model and shown to systemically deplete MDSCs, resulting in increased T and NK cell infiltration into the tumor environment (21). The MDSC-targeting peptide was found *via* phage display screening to target S100A8/A9, a calcium binding protein that has been shown to impair neutrophil and leukocyte function, reduce cytokine secretion, and inhibit antigen presentation on antigen-presenting cells (22–26). S100A8/A9 is known to be highly present on myeloid-lineage cells following inflammation, such as MDSCs (27). Based on their efficacy in the cancer model (21), these MDSC-targeting nanoparticles could be a useful immunomodulatory therapeutic in conjunction with a local treatment strategy to improve healing outcomes following musculoskeletal trauma.

Therefore, the objective of this study was to use a novel, non-surgical nanoparticle approach targeting the *systemic* immune response to investigate how immunomodulation of MDSCs impacts regenerative outcomes following delayed treatment in a model of composite musculoskeletal trauma containing both a critically sized bone defect and a volumetric muscle injury. While the composite trauma model has been previously established and investigated, here an additional component of delayed treatment was added, representing the clinical delay in treatment of a non-union defect and the biological changes that occur during that period. Along with a systemic immunomodulatory treatment, we utilized local administration of bone morphogenetic protein-2 (BMP-2), an osteoinductive protein with FDA approval for use in treating spinal defects. We hypothesized that the MDSC-targeting Janus nanoparticles in conjunction with a local BMP-2 treatment strategy would decrease systemic levels of MDSCs, resulting in improved bone regeneration compared to BMP-2 treatment alone. We also longitudinally monitored cytokine and immune cell populations systemically from the blood to identify predictive biomarkers of treatment outcomes and to characterize the immunological factors that contribute to the development of non-unions and poor healing. Single cell RNA sequencing was also conducted to better understand the role of the immunosuppressive MDSC populations in a model of composite trauma. A better understanding of the immunological markers associated with non-union progression and poor healing could be essential for facilitating patient-specific immunomodulatory care that optimizes and improves the current standard of care.

## Materials and Methods

### Nanofiber Mesh and Alginate BMP-2 Preparation

Nanofiber mesh and alginate hydrogel constructs were prepared as previously described (28). Briefly, to make the polycaprolactone (PCL) nanofiber meshes, a 90:10 solution of 1,1,1,3,3,3-Hexafluoro-2-propanol, 99+% (HFP; Sigma Aldrich) and *N,N*-Dimethylformamide, anhydrous, 99.8% (DMF; Sigma Aldrich) was prepared. PCL was added to a concentration of 12% w/v in the 90:10 solution of HFP:DMF and allowed to dissolve overnight. Meshes were then electrospun to an approximate thickness of 500 µM and a laser cutter was used to create 0.7 mm diameter perforations throughout the mesh. UV cure adhesive (DYMAX) was used to glue the meshes to a rolled inner diameter of 4.5 mm and a length of 12 mm. Meshes were then sterilized by Ethylene Oxide and then stored in alpha-MEM at 4°C until use. To make the alginate hydrogel constructs, a 3% w/v alginate solution was created by dissolving RGD-functionalized alginate (FMC Biopolymer) into sterile alpha-MEM (Corning). The solution was then mixed with 0.1% rat serum albumin (RSA) in 4 mM HCl containing 2.5 µg of BMP-2 per defect and cross-linked in an excess of calcium sulfate by thorough mixing. The alginate gels were stored in syringes at 4°C until use.

### Fabrication of MDSC-Depleting Nanoparticles

Synthetic nanoparticle antibodies (SNAbs) targeting MDSCs were prepared as previously described using a solid phase synthesis method (21). Briefly, aminomethyl ChemMatrix resin (SigmaAldrich) was hydrated and then reacted with a disulfide NHS biotin-functionalized crosslinker (sulfo-NHS-SS-biotin, ThermoFisher Scientific). After washing off unbound crosslinker, 30 nm gold Janus nanoparticles (Nanohybrids) were reacted overnight. Following particle binding, reaction vessels were again washed to remove unbound particles and then reacted with Tris(2-carboxyethyl)phosphine (TCEP, ThermoFisher Scientific) overnight to cleave particles from the resin at the disulfide within the crosslinker. Janus particles were then dialyzed against phosphate buffer to remove excess TCEP. A biotin-functionalized MDSC targeting peptide (G3-Biotin, WGWSLSHGYQVK(K-Biotin)) and an SMCC- functionalized Fc-mimicking peptide (Pep33-SMCC, AQVNSCLLLPNLLGCGDDK-(K-SMCC)) were reacted with the Janus particles sequentially overnight at 4°C with washing in between peptide additions. Final particles were collected *via* centrifugation, sterile filtered, and resuspended in 1X sterile PBS until injection.

### Surgical Procedures

#### Infection Model

All animal care and experimental procedures were approved by the Veterans Affairs Institutional Animal Care and Use Committee (IACUC) and carried out according to the guidelines. The infection model is used as a source of MDSCs for *in vitro* experiments. This established, published trauma model has been shown to result in a large, sustained increase in MDSCs that can be isolated repeatedly and easily through blood draws. Surgical procedures were carried out as previously described (20). Briefly, unilateral 2.5 mm segmental defects were created in the mid-diaphysis of the femur using 12-week old female Sprague Dawley rats (Charles River Labs) using a Gigli wire saw (RISystem). Twelve-week-old rats were selected because they are skeletally mature and because approximately12–15 weeks is when female Sprague Dawley rats reach their maximum growth in weight and femur size. Defects were then stabilized with an internal modular fixation plate. A collagen sponge inoculated with 10^7^ CFU of *Staphylococcal aureus* was then placed in the defect site. The 10^7^ CFU was selected based off of prior work showing that at this dosage there is sustained, elevated levels of MDSCs and a local infection is maintained with minimal hardware failure or abscess formation through 8 weeks (20). The incision site was sutured closed with absorbable 4-0 sutures and wound clips. Subcutaneous administration of slow-release buprenorphine (0.03 mg/kg; 1 ml/kg) was used as an analgesic.

#### Composite Trauma Model

All animal care and experimental procedures were approved by the University of Oregon IACUC and carried out according to the guidelines. Surgical procedures were carried out as previously described (19). Briefly, unilateral 8 mm femoral segmental defects with 8 mm diameter, full-thickness quadriceps muscle defects were created in 14-week-old female Sprague Dawley rats (Charles River Laboratories) using an oscillating saw and a biopsy punch. Fourteen-week-old rats were selected because they are skeletally mature and because approximately 12–15 weeks is when female Sprague Dawley rats reach their maximum growth in weight and femur size. Defects were stabilized with a polysulfone, radiotranslucent internal fixation plate and left untreated for 8 weeks at which point animals underwent an additional surgery to treat the bone defect, representing the clinical delay in treatment of a non-union. The bone defect site was cleared and 2.5 µg of BMP-2 (Pfizer) was added *via* the nanofiber mesh alginate hybrid delivery system. The muscle defects were left untreated. At 9 weeks post-injury, a subset of animals received no synthetic nanoparticle antibodies (SNAbs) (*n* = 11), and another subset of animals received arterial injections of a single (*n* = 5) or double dose (*n* = 5) (1x SNAb or 2x SNAb) of SNAbs. Animals were euthanized at 20 weeks post-injury. Histology was conducted on a subset of rats (no SNAbs, *n* = 2; 1x SNAb, *n* = 1; 2x SNAb, *n* = 1), and mechanical testing was conducted on the remaining rats (no SNAbs, *n* = 9; 1x SNAb, *n* = 6, 2x SNAb, *n* = 4). Supplementary Figure S1 shows a timeline of the study for the SNAb and non-SNAb treated groups.

### Immune Characterization

Blood was drawn from the tail artery of rats *via* Vacuette blood collection needles (Greiner Bio-One) at various timepoints post-injury and treatment including Day 3, Weeks 1, 4, 7, Week 8 Day 5, Week 9 Days 1 and 2, Weeks 10, 12, 16, and 20 to monitor immune cell populations and chemokine and cytokine levels over time. The Day 3 timepoint was selected to observe the early immune response to injury, whereas the Week 8 Day 5 response was selected to observe immune function prior to SNAb treatment at Week 9. Approximately 300 µL of blood was collected into Microtainer lithium-heparin coated tubes (BD) for FACS analysis and approximately 150 µL of blood was collected into Microvette serum collection tubes (Kent Scientific) for serum analysis. For whole blood analysis, red blood cells were lysed using 1X RBC lysis buffer (eBioscience) according to manufacturer's instructions. Cells were then fixed and resuspended in FACS buffer containing 2% FBS in PBS. Cells were first incubated with anti-rat CD32 to prevent non-specific antibody binding and then stained for various immune cells including T cells (CD3+), cytotoxic T cells (CD3 + CD8+), helper T cells (CD3 + CD4 + FoxP3−), regulatory T cells (Tregs, CD3 + CD4 + FoxP3+), B cells (B220+), monocytes (CD11b + CD68+), and MDSCs (CD11b + His48+). Data were collected using a BD Accuri C6 flow cytometer and analyzed with FlowJo software with gates set based on FMO controls allowing <1% noise. The gating strategy can be found in Supplementary Figure S2, and a list of antibodies and dilutions can be found in Supplementary Table S1. For cytokine and chemokine analysis, serum was collected after allowing blood to clot for approximately 2.5 h at 4°C and then spinning down samples at 1,000 g for 10 min. The supernatant was collected and stored at −20°C until multiplexed chemokine and cytokine analysis (Luminex) was conducted using Milliplex MAP Rat Cytokine and Chemokine Magnetic kits (Millipore Sigma). Data was collected on the MAGPIX Luminex instrument and analyzed with the median fluorescent intensity values with background subtracted.

### Bone Regeneration

Longitudinal bone regeneration was qualitatively assessed with radiographic imaging using an UltraFocus digital radiography system (Faxitron), and quantitatively assessed with micro-computed tomography using the vivaCT80 (Scanco Medical) both *in vivo* at six weeks post-BMP-2 treatment and *ex vivo* at twelve weeks post-BMP-2 treatment. The middle region of the defect was analyzed (∼6.5 mm) with a 55-kVp voltage and a 145-µA current, and the voxel size was set to 48.5 µm for *in vivo* scans and 24 µm for *ex vivo* scans. Newly regenerated bone was segmented using a threshold corresponding to 50% of the average bone mineral density of native cortical bone. Functional bone regeneration was also assessed quantitatively using biomechanical testing to evaluate torsional stiffness and failure strength. Femurs were excised and potted in Wood's metal and then tested to failure in torsion at a rotation rate of 3 degrees per second using the ELF3200 testing system (TA Instruments). Failure strength was determined as the peak torque within the first 90 degrees of rotation and torsional stiffness was calculated by finding the slope of the linear region before failure in the torque-rotation plot.

### *In Vitro* Co-Culture Assay

SNAbs were tested *in vitro* in a co-culture assay containing MDSCs (target cells) and macrophages (effector cells). The SNAbs require the presence of both target and effector cells, similar to antibodies, in order to elicit specific killing of the target cells (MDSCs). The MDSCs were enriched from the blood of infected trauma rats. Blood was drawn *via* the rat tail artery and collected into Microtainer lithium-heparin coated tubes (BD). Blood was pooled and then red blood cells were lysed using 1X RBC lysis buffer (eBioscience). MDSCs were then enriched using magnetic activated cell sorting (Miltenyi) according to the manufacturer's instructions with His48 as the sorting marker. Cells were then washed, counted, and resuspended in RPMI 1,640 media. One week prior to MDSC isolation, macrophages were isolated from the bone marrow of one naïve, healthy female Sprague Dawley rat. The femur and tibia from both legs were excised and a syringe and 20G needle were used to flush the bone marrow cavities with 1X PBS. Cells were then run through a 40 µM cell strainer and red blood cells were lysed using 1X RBC lysis buffer (eBioscience). Cells were then washed, counted, resuspended in RPMI 1,640 media, and plated at 5e^5^ cells/ml in a petri dish with 20 ng/ml recombinant rat macrophage colony stimulating factor (M-CSF, Peprotech). Cells were cultured for 7 days with media changes on Days 3 and 6 and flow cytometry was used to confirm successful differentiation on Day 7 (Supplementary Figure S3). Cells were plated at a 1:10 ratio of MDSCs to macrophages in a 96 well plate with 500,000 cells per well. In SNAb treated wells, 1e^10^ SNAbs were added in 50 µL of 1X PBS. 50 µL of 1X PBS was added to untreated, control wells. Following a 24-hour co-culture period, cells were collected and single cell RNA sequencing was conducted on SNAb-treated cells enriched for MDSCs to better understand the impacts of SNAb treatment on rat MDSCs. Untreated, pre-SNAb treated cells were used as a control.

### Single Cell RNA Sequencing

Rat PBMCs were isolated from both naïve rats (*n* = 3) and composite trauma model rats (*n* = 3) *via* the tail artery using Vacuette blood collection needles (Greiner Bio-One). Blood was isolated from the composite trauma model rats at Day 5 post-injury and pooled together for subsequent analyses. Cell number and viability was confirmed to achieve the target 5,000 barcoded cells. Single cell RNA sequencing (scRNAseq) was performed using 10X Genomics Single Cell 3′ Solution (version 3.1), according to the manufacturer's instructions (protocol rev C). Libraries were sequenced on Nextseq500 (Illumina) and data were de-multiplexed, aligned, and counted using Cell Ranger version 3.1.0 (10X Genomics). Data were analyzed using the R package Seurat (https://satijalab.org/seurat/) developed by the Satija Lab and is specifically designed for quality control, analysis, and exploration of single cell RNA sequencing data. The package utilizes Louvain clustering which modularly optimizes large networks to detect communities using an unsupervised algorithm. These clusters can then be visualized using uniform manifold approximation and projection (UMAP) which dimensionally reduces the data into 2 dimensions, UMAP-1 and UMAP-2, or using t-distributed stochastic neighbor embedding (t-SNE) which dimensionally reduced the data into two dimensions, tSNE-1 and tSNE-2. Quality control metrics were utilized and excluded cells with mitochondrial gene percentage greater than 20% or cells with feature counts below 200 or above 2,500.

### Statistical Analysis

Statistical significance for quantitative results was determined using appropriate parametric or non-parametric statistical tests and significance was determined by *p* values less than 0.05. Statistical analyses were performed using GraphPad Prism 9.0 software (La Jolla, CA, USA).

## Results

### MDSC Depletion *in Vitro* from Rat Trauma-Derived Cells

Initial experiments were conducted using MDSCs derived from a rat segmental defect model containing a biomaterial-based *Staphylococcus aureus* infection. This model was selected due to previous work showing high levels of MDSCs as early as 3 days post-surgery which stay elevated for several weeks post-injury (20). The high levels of MDSCs derived from this model allows for *in vitro* evaluation of MDSC depletion using the synthetic nanoparticle antibodies (SNAbs). The SNAbs are 30 nm gold Janus nanoparticles which contain MDSC-targeting peptides conjugated to one domain and Fc-mimicking peptides conjugated to the other domain, allowing the nanoparticles to mimic the structure and function of antibodies, resulting in antibody-mediated killing of the MDSC target cells. Previous work has confirmed the ability of SNAbs to deplete MDSCs systemically in a mouse tumor model (21); however, reduction of MDSC levels by SNAbs in rats has not previously been evaluated.

**Figure 1:**
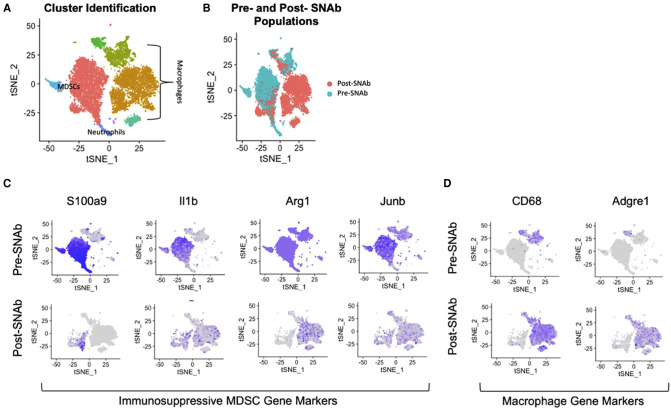
Single cell RNA sequencing of MDSC depletion *in vitro.* (**A**) Clustering of integrated tSNE plots from the SNAb-treated and untreated cells enriched for MDSCs. (**B**) Overlay of tSNE plots from SNAb-treated and untreated groups showing differences in cell populations. (**C**) Gene expression for immunosuppressive MDSCs gene markers (*S100a9*, *IL1b*, *Arg1*, and *Junb*) pre- and post-SNAb treatment. (**D**) Gene expression for macrophage gene markers (*CD68* and *Adgre1*) pre- and post-SNAb treatment.

SNAbs were tested *in vitro* in a co-culture assay containing MDSCs and macrophages. The MDSCs were enriched from the blood of infected trauma rats using magnetic activated cell sorting and the macrophages were differentiated from the bone marrow of healthy, naïve rats using M-CSF stimulation. Following a 24-hour co-culture period, single cell RNA sequencing was conducted on SNAb-treated cells enriched for MDSCs to better understand the impacts of SNAb treatment on rat MDSCs. Untreated, pre-SNAb treated cells were used as a control. Integration and clustering of both the treated and untreated groups shows the presence of MDSCs, macrophages, and neutrophils (Figure 1A). An integrated overlay of tSNE projections from the SNAb treated and untreated groups shows depletion of the MDSC clusters (Figure 1B), which was also confirmed *via* flow cytometry (21). In addition, expression of immunosuppressive gene markers known to be highly expressed in MDSCs, including *S100a9*, *IL1b*, *Arg1* (arginase 1), and *Junb* (a transcription factor), are all significantly decreased post-SNAb treatment (Figure 1C). The presence of the macrophage clusters resulting from the co-culture assay are confirmed with the expression of macrophage gene markers, including *CD68* and *Adgre1* (Figure 1D). These results demonstrate that SNAbs are capable of functionally depleting MDSCs, both in cell numbers and *via* decreased immunosuppressive gene expression.

### Systemic Immune Cell Populations are Altered in Response to Injury and SNAb Treatment

**Figure 2:**
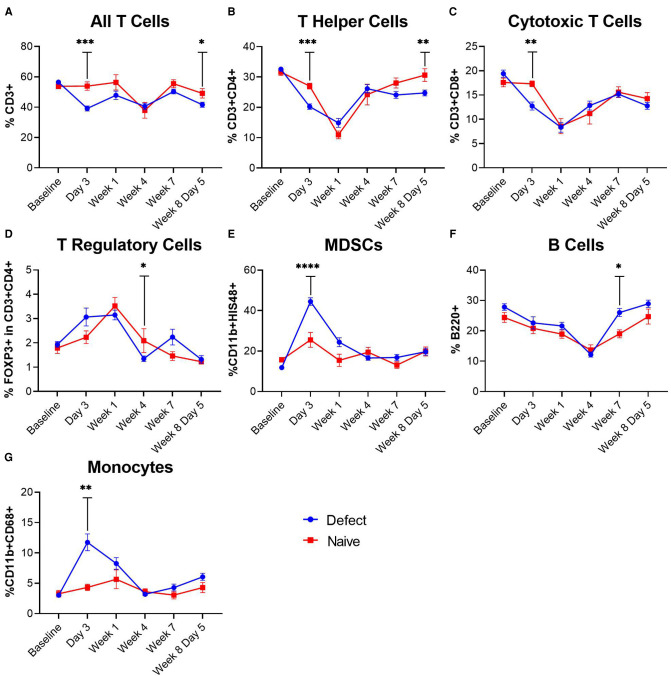
Characterization of circulating immune cells after injury (Day 0) and treatment (Week 8). (**A**–**G**) Significant changes in immune cell populations were observed in rats after injury. T cells, T helper cells, and cytotoxic T cells were significantly decreased in injured rats (**A**–**C**), while MDSCs and monocytes were significantly elevated (**E,G**). Data are mean ± SEM, *n *= 7 to 13 per group. *P < 0.05; **P < 0.01; ***P < 0.001; ****P < 0.0001 as indicated.

Blood was collected at the baseline (prior to injury) and at various timepoints post-injury, post-BMP-2 treatment, and post-SNAb treatment to assess the systemic immune response. At day 3 post-injury, MDSCs and monocytes were significantly elevated while T cells and T helper cells were significantly depressed in the composite defect group compared to naïve controls (Figure 2). T cells and T helper cells were also significantly decreased following BMP-2 treatment at week 8 day 5, although other immune cells did not show significant changes at that timepoint, in particular MDSCs, in contrast to observations made in previous studies and cohorts (18, 19). In fact, in this study, the MDSC levels were back to pre-trauma levels at the time of SNAb treatment.

**Figure 3:**
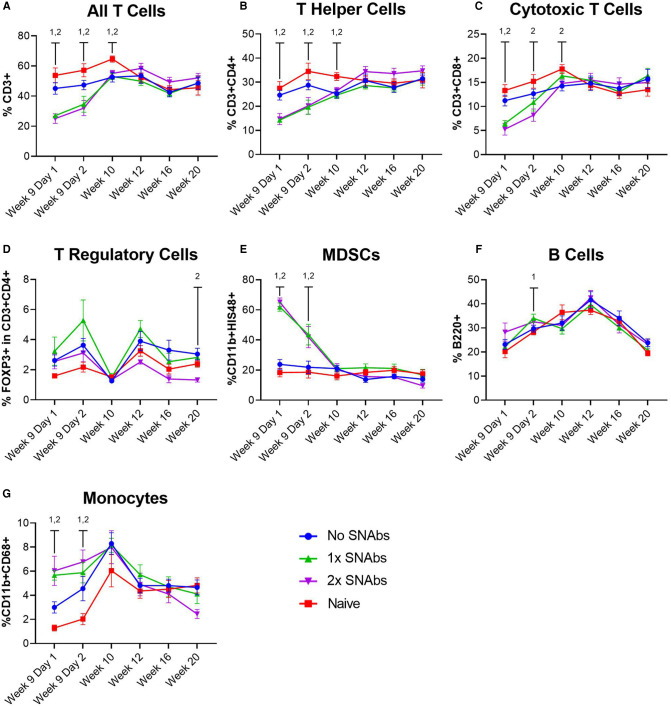
Characterization of circulating immune cells after treatment with synthetic nanoparticle antibodies (SNAbs). (**A**–**G**) Significant changes in immune cell populations were observed in rats at multiple timepoints after SNAb injections. T cells, T helper cells, and cytotoxic T cells were significantly decreased in SNAb-injected rats (**A**–**C**), while MDSCs and monocytes were significantly elevated (**E,G**). Data are mean ± SEM, *n *= 7 to 13 per group. 1,2 correspond to significant differences between the naive group and the 1x SNAbs group (1) or the 2x SNAbs group (2), with *P* < 0.05.

**Figure 4:**
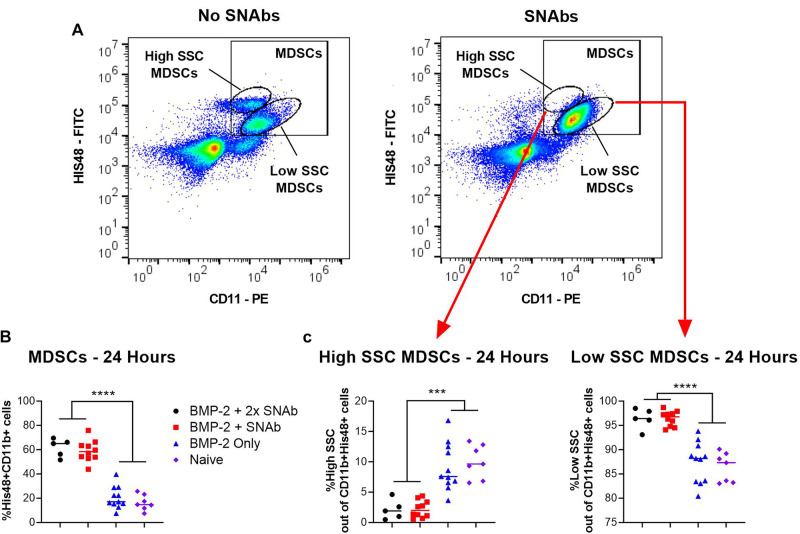
MDSCs at 24 h post-SNAb treatment in the composite trauma model. (**A**) Flow cytometry dot plots showing two MDSC sub-populations. Systemic levels of total MDSCs (**B**) and the two MDSC subsets (**C**) at 24 h post-SNAb treatment in the composite trauma model. Significance was determined using a one-way ANOVA. ***P < 0.001; ****P < 0.0001 as indicated; *n *= 5 to 11 per group.

At 1 and 2 days post-SNAb treatment (Week 9 Days 1 and 2), both SNAb-treated groups (both the single and double dose groups) showed significantly elevated levels of MDSCs compared to the BMP-2 only and naïve groups (Figure 3). However, looking at the MDSC population *via* flow cytometry reveals two distinct MDSC sub-populations – a high side scatter population and a low side scatter population (Figure 4A). MDSCs are known to have both monocytic and granulocytic subsets in humans and mice (29), and the observed MDSC sub-populations in this rat data likely parallel these known subsets in other species. The two MDSC sub-populations respond differently to SNAb treatment. On one hand, the high side scatter MDSCs were significantly decreased at 24 h post-treatment in both SNAb-treated groups, whereas low side scatter MDSCs were significantly increased at 24 h post-treatment in both SNAb-treated groups (Figure 4C). However, despite a significant decrease in the high side scatter MDSCs following SNAb treatment, there is still an overall increase in total MDSCs (Figure 4B) due to the larger size of the low side scatter MDSC sub-population. In addition to changes in the MDSC populations post-SNAb treatment, significant differences in T cells, T helper cells, cytotoxic T cells, and cytokine levels were also observed at Week 9 Days 1 and 2 post-treatment, demonstrating the wide-ranging effects of SNAbs on the systemic immune response (Figure 3, Supplementary Figure S4). Notably, T cells and the T helper cell and cytotoxic T cell subsets were significantly decreased at Days 1 and 2 post-SNAb treatment (Figure 3).

### SNAb Treatment Results in Decreased Functional Bone Regeneration

**Figure 5:**
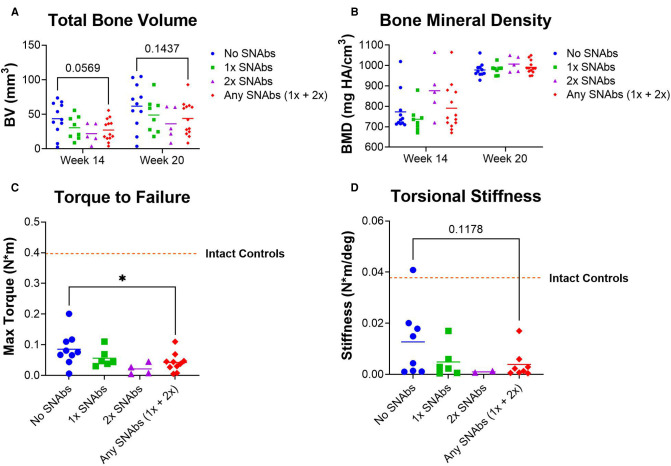
Functional regeneration may be impaired following treatment with synthetic nanoparticle antibodies (SNAbs). (**A,B**) Total bone volume (**A**) and bone mineral density (**B**) of newly formed bone, as quantified by *in vivo* µCT at week 14 and ex vivo µCT at week 20. (**C,D**) Mechanical strength (**C**) and stiffness (**D**) of regenerated femurs as quantified by ex vivo torsional testing to failure at week 20. The dotted lines indicate mechanical properties of intact bone. *P < 0.05 as indicated.

Functional bone regeneration was assessed by radiographic imaging and micro-computed tomography (uCT) at six weeks (Week 14) and twelve weeks (Week 20) post-BMP-2 treatment as well as biomechanical testing twelve weeks post-BMP-2 treatment. No significant differences were observed in the bone volume or mechanical properties between the BMP-2 only group and either SNAb-treated group individually (Figure 5). However, when comparing the mechanical strength of the femurs treated with BMP-2 only to all SNAb-treated femurs, the BMP-2 only group had significantly increased failure strength compared to the SNAb-treated rats (Figure 5C). Furthermore, the BMP-2 only group (no SNAbs) had higher peak bone volume and higher peak mechanical properties compared to any of the SNAb-treated groups (Figures 5A, C, D). Although not statistically significant, the average bone volume, failure strength, and torsional stiffness all decrease with each increasing dose of SNAbs. These data are further supported by representative 3D reconstructions and histology of the defect sites (Supplementary Figure S5). Further studies with increased power are needed to identify if there is a dose-dependent response to SNAbs that results in decreased functional bone regeneration.

### Systemic Immune Cell Populations Correlate with Bone Regeneration

**Figure 6:**
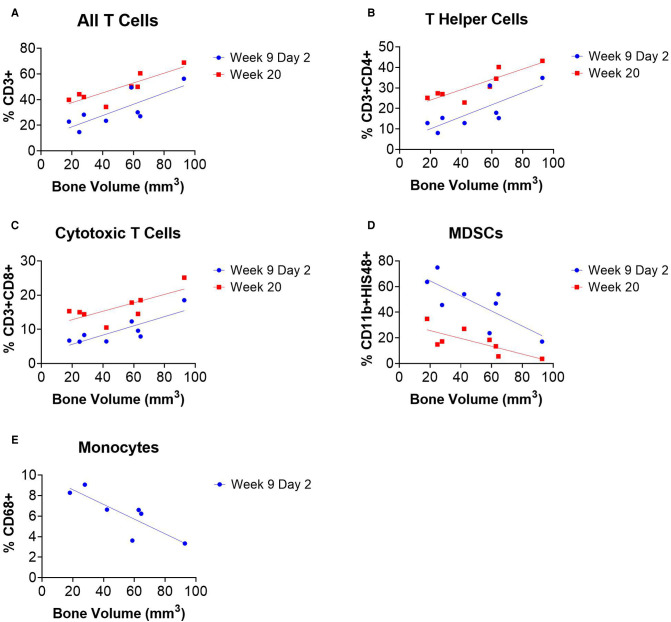
Circulating immune cells correlate with bone regeneration in rats treated with synthetic nanoparticle antibodies (SNAbs). (**A**–**E**) Immune cell populations from peripheral blood at multiple timepoints significantly correlate with week 20 bone volumes as measured by ex vivo µCT. T cells, T helper cells, and cytotoxic T cells were positively correlated with bone regeneration (**A**–**C**), while MDSCs and monocytes were negatively correlated (**D,E**). Pearson correlation analyses performed, *n *= 7–8 per time point, slope of linear regression significantly nonzero for all data shown (*P* < 0.05).

**Figure 7:**
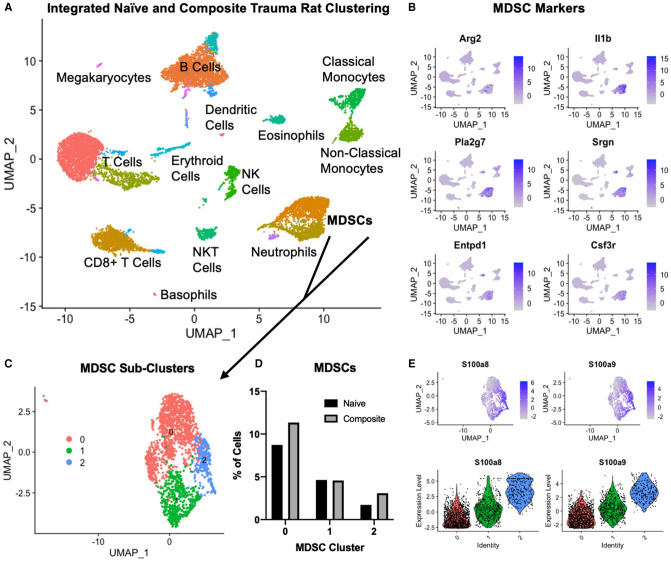
Single cell RNA sequencing in the composite trauma model. (**A**) Integrated clustering of PBMCs from naïve and composite trauma rats at 5 days post-injury. (**B**) Gene expression of MDSC gene markers. (**C**) MDSC clusters identified in (**A**) were re-integrated and re-clustered for more in-depth analysis. Three subclusters were identified. (**D**) Comparison of MDSC percentages for clusters 0, 1, and 2 in composite trauma model rats versus naïve rats. (**E**) Density plots (top) and violin plots (bottom) of *S100a8* expression (left) and *S100a9* expression (right).

Linear regression analyses revealed correlations between systemic immune cell populations and endpoint bone volume of SNAb-treated rats in the single dose SNAb-treated group (Figure 6, Supplementary Table S2). Specifically, T cells, T helper cells, and cytotoxic T cells measured 48 h after SNAb injections (Week 9 Day 2) and at the endpoint (Week 20) were found to correlate positively with healing (Figures 7A–C), while MDSCs and monocytes measured 48 h after SNAb injections (Week 9 Day 2) and MDSCs measured at the endpoint (Week 20) were found to correlate negatively with healing (Figures 6D, E). In addition, when looking at all rats, MDSCs negatively correlated with endpoint bone volume at Week 9 Days 1 and 2 and T cells, T helper cells, and cytotoxic T cells all positively correlated with endpoint bone volume at Week 9 Days 1 and 2 (Supplementary Figure S6). These results agree with observations made in our previous studies of bone-only injury models, where T cells and T helper cells correlated positively with healing and MDSCs and monocytes correlated negatively (18).

### Single Cell RNA Sequencing Reveals Differing MDSC Gene Expression Patterns

In order to better understand why the SNAbs only targeted one subset of MDSCs, we conducted single-cell RNA sequencing (scRNAseq) on PBMCs isolated from the composite trauma model rats at 5 days post-injury. PBMCs from naïve rats were also isolated and data from both the composite trauma and naïve rats were integrated, clustered, and analyzed (Figure 7A). Clusters were identified with marker genes for each immune cell type. Gene expression of MDSC gene markers, including Arg2, Il1b, Pla2g7, Srgn, Entpd1, and Csf3r, was elevated in the two clusters marked MDSCs (Figures 7A, B). To further analyze the MDSC populations, these clusters were isolated, re-integrated, and re-clustered, resulting in 3 MDSC sub-clusters (Figure 7C). Cluster 0 accounts for the highest percentage of MDSCs, followed by Cluster 1, and lastly by Cluster 2. Comparing the percentages of MDSCs in the composite trauma rats versus the naïve rats shows an increase in MDSCs in Clusters 0 and 2 only in the composite trauma rats (Figure 7D). Because these two clusters exhibit a marked increase in MDSCs compared to naïve rats, these are likely the two most important clusters to target with the SNAbs. In addition, because Cluster 0 accounts for a much higher percentage of MDSCs than Cluster 2, Cluster 0 may be the most important target in order to achieve a therapeutic effect. The MDSC-depleting SNAbs target S100A8/A9 on the surface of MDSCs, so we next investigated S100A8/A9 expression by looking at the genes for these proteins, *S100a8* and *S100a9*. Please note that S100A8/A9 will be used to refer to the protein, whereas *S100a8/a9* will be used to refer to the gene. *S100a8* and *S100a9* gene expression is highest in Cluster 2, with some expression in Cluster 1, and minimal expression in Cluster 0 (Figure 7E). While gene expression levels are not necessarily equivalent to protein levels, these differences in *S100a8* and *S100a9* gene expression could account for the MDSC depletion differences observed in the two different MDSC sub-populations *via* flow cytometry. Improved markers are needed in order to ensure successful targeting of all MDSC subsets in the composite trauma model.

## Discussion

Despite significant advances in trauma care and management, orthopedic surgeons still need improved strategies to address complicated musculoskeletal trauma, especially for more challenging cases, such as non-unions, concomitant volumetric muscle loss, or bone infections. Prior work in our lab has highlighted the role of systemic immune dysregulation and immunosuppression following trauma as a negative predictor of local healing outcomes in both non-union and composite trauma models (18, 19). Most notably, in the non-union model, an inverse correlation at 1-week post-treatment was observed between circulating MDSC levels and endpoint bone volume (18), suggesting that immunomodulatory therapeutics that target and deplete MDSCs could potentially improve healing outcomes. This study seeks to address this question by evaluating the ability of MDSC-targeting synthetic nanoparticle antibodies (SNAbs) to deplete MDSCS, restore systemic immune homeostasis, and alter the local healing environment to improve functional bone regeneration. The relationship between the systemic and the local immune response has been well established, but more recently, there has been more work done investigating the therapeutic relationship between these two environments. For example, one study in cancer immunotherapy demonstrated that systemic immunity is required for successful anti-tumor immune therapy, and another study showed that systemic immune homeostasis is altered by local biomaterial scaffolds for local tissue regeneration (30, 31). Based on the association between the local and systemic immune environments, we posit that consideration of immune homeostasis and a permissive, pro-healing immune environment at both the local and systemic levels is necessary for regenerative medicine strategies to reach their full potential.

Prior to a full investigation of the impact of SNAb treatment on healing in a composite trauma model, *in vitro* investigations of SNAbs were conducted using MDSCs derived from an infected trauma model. *In vitro*, SNAbs resulted in a decrease in MDSCs as well as a decrease in immunosuppressive gene expression, including *S100a9*, *Il1b*, *Arg1* (arginase), and *Junb*. *S100a9* is of particular interest because the S100A9 protein is upregulated on the surface of MDSCs, secreted by MDSCs, and is one of the target proteins of the MDSC-targeting peptide on the SNAbs. Along with arginase (*Arg1*), S100A9 plays a role in T cell immunosuppression. S100A9 induces upregulation of PD-1/PD-L1, leading to T cell exhaustion and apoptosis (25). Arginase on the other hand suppresses T cell functions by depleting L- arginine, an essential amino acid for T cell receptor (TCR) signaling (32). Il-1b (*IL1b*) is another important MDSC-associated cytokine which recruits and activates MDSCs through the IL-1R/NF-kB pathway, resulting in further accumulation of MDSCs (33, 34). Lastly, Junb (*Junb*), a transcription factor in the activating protein (AP-1) family, is another marker of aberrant immune cell responses and is involved in the immune cell activation program in MDSCs (35). Significant reduction in expression of these immunosuppressive and MDSC- associated genes shows promise that SNAbs can be utilized to successfully alter an immunosuppressive environment by targeted killing of MDSCs.

Given these positive preliminary results, the impact of SNAbs on the systemic immune response and functional bone regeneration was investigated in a composite trauma model. This study demonstrated multiple changes to systemic immune cell populations in response to both composite injury and immunomodulation treatment. The elevated levels of MDSCs and monocytes coupled with depressed T cells, T helper cells, and cytotoxic T cells observed after injury indicated the development of trauma-induced systemic immune dysregulation and immunosuppression. Furthermore, depressed levels of T cells and T helper cells were observed following revision surgeries to treat with BMP-2, similarly to the response following the initial injury surgery. These results also agree with observations made in previous studies and models including a delayed treatment non-union model and composite trauma model (18, 19). Following the injury surgery, a decrease in T cells and an increase in MDSCs was observed. However, following BMP-treatment (revision) surgery, only a decrease in T cell levels was observed with no increase in MDSCs, which was an unexpected result. MDSCs were only significantly elevated at day 3 post-injury surgery, so a similar spike in MDSCs at day 3 post-revision surgery may have occurred but was not able to be observed at the 5-day post-revision surgery timepoint used in this study.

Although MDSC depletion was observed *in vitro*, SNAb treatment in the pre-clinical composite trauma model resulted in an overall increase in total MDSCs. Two distinct sub-populations of MDSCs were observed that made up the total number of MDSCs – a high side scatter population and a low side scatter population. It was noted that while MDSCs significantly increased in the larger sub-population of low side scatter MDSCs, MDSCs actually significantly decreased in the much smaller high side scatter sub-population. These two observed MDSC sub-populations likely mirror monocytic and granulocytic MDSC sub-populations that have been observed in humans and mice (29). Single cell RNA sequencing analysis revealed differences in *S100a8/a9* gene expression, which is the gene responsible for producing the target molecule of the MDSC-targeting peptide used on the SNAbs. S100A8/A9 is a calcium-binding protein that participates in cytoskeleton rearrangement and is released during inflammation to stimulate leukocyte recruitment and induce cytokine secretion (27, 36). Despite its role in inflammation, S100A8/A9 is also known to play a significant immunosuppressive role including by impairing neutrophil infiltration, reducing cytokine secretion, inhibiting antigen presentation on antigen presenting cells, and suppressing leukocyte adhesion and migration (22–26). While S100A8/A9 is known to be highly expressed on myeloid-lineage cells following inflammation, such as MDSCs, it is also highly expressed in neutrophils, making up approximately 45% of cytoplasmic proteins (27). Based on the wide and varied roles of S100A8/A9, expression and secretion can change during inflammation and may vary widely in differing causes of inflammation, such as that caused by composite trauma versus infection or cancer. *S100a8/a9* gene expression was highest in the smallest MDSC sub-cluster and lowest in the largest MDSC sub-cluster, suggesting that effective targeting may only have occurred for the smallest MDSC sub-cluster. This is supported by flow cytometry data showing successful depletion of the smaller, high side scatter MDSC sub-population. An improved understanding of S100A8/A9 and how the inflammatory and anti-inflammatory duality impact the systemic immune response over time in different cases of inflammation will be essential for robust and consistent SNAb targeting of immunosuppressive MDSCs. In addition, an improved understanding of MDSC sub-populations and identification of new surface targets that are highly expressed in all MDSC sub-populations could drastically improve MDSC targeting and depletion.

SNAb treatment resulted in a significant increase in MDSCs at Week 9 Days 1 and 2 post-treatment as well as a significant decrease in immune effector cells, including T cells and the T helper cell and cytotoxic T cell subsets. In addition to these cellular changes, multiple significant differences in cytokine levels were observed at both Week 9 Days 1 and 2. These data suggest that SNAbs have the capability to significantly alter multiple aspects of the systemic immune response on both a cellular and protein level. While these cellular and protein differences were not sustained past Week 9 Day 2, we still observed significant correlations between multiple immune cell types at Week 20 and endpoint bone volume in the SNAb treated rats. This suggests that the temporal changes in immune cell populations and cytokine levels at 24- and 48- hours post-SNAb treatment may still have had long-term effects, even 11 weeks later. Further studies are needed to better understand the mechanisms of SNAb treatment on the systemic immune response and if multiple SNAb treatments are needed in order to achieve more robust responses.

SNAb depletion of only the high side scatter MDSC subset in the composite trauma model did not improve local healing, likely due to the significant increase in total MDSCs that overshadowed the depletion in the high side scatter sub-population. SNAb treatment appeared to worsen bone repair; however, because SNAb treatment resulted in an increase in total MDSCs, this is consistent with our previous data showing a negative correlation between MDSCs and healing. When comparing all SNAb treated rats to rats that did not receive SNAbs, the failure strength in torsion was significantly decreased, suggesting poor functional regeneration. Due to aberrant SNAb targeting and the large increase in the low side scatter MDSCs following SNAb treatment, it is not surprising to see negative effects on bone regeneration. This is further supported by linear regression analyses which demonstrated multiple immune cell populations in SNAb-treated rats that correlated either positively or negatively with healing just 48 h after treatment. These correlations mirror those found in previous studies of segmental defect injuries treated with BMP-2 only (18), suggesting that the detrimental effects of the SNAbs on healing in this study were likely immune-mediated. Of note, monocytes also correlated negatively with endpoint bone volume, similar to MDSCs. MDSCs and monocytes are both myeloid-lineage cells, and therefore may share similar surface markers, such as the target molecule of the SNAbs. Further work is needed to understand how MDSC-depleting SNAbs interact specifically with monocytes and macrophages, and why monocytes also negatively correlate with endpoint bone volumes. In addition, improved MDSC targeting may be necessary to achieve robust depletion of both subsets of MDSCs to restore immune homeostasis and positively impact regeneration.

It should be addressed that this study included no unmodified particles or scrambled peptide modified particles as controls due to animal limitations and limitations with reagent scale-up. A large sample size was needed to account for the non-responders and responders within each treatment group, and at *n* = 10 per group, multiple control groups were not physically feasible. However, previous work with SNAbs in a murine cancer model did utilize irrelevant peptide and unmodified particles as controls, and these did not elicit similar responses to the MDSC-targeting SNAbs (21). It should also be addressed that BMP-2 has been shown to have immunomodulatory effects, including macrophage stimulation and upregulation of cytokines important for cell recruitment and angiogenesis (37). Because of this, all rats were treated with BMP-2 to eliminate any potential confounding factor due to local immunological changes. It is not expected that systemic MDSC-depleting SNAbs alone can alter local bone healing, and therefore, this necessitates a local treatment strategy, which in this case utilizes BMP-2. We posit that that consideration of immune homeostasis and a permissive, pro-healing environment at both the local and systemic levels is necessary for regenerative medicine strategies to reach their full potential. BMP-2 was selected as the local treatment strategy because it is a potent osteoinductive growth factor with FDA approval in select applications including spinal fusions and some tibia fractures (38). In addition, pre-clinical and clinical studies over the past decade have shown significant promise for BMP-2 treatment in long bone fractures (39).

In conclusion, this study examined the effects of a novel, non-surgical nanoparticle approach for modulating MDSCs and the systemic immune response in a composite injury model of trauma. This approach was successful in depleting MDSCs and associated immunosuppressive genes *in vitro* using cells derived from an infected trauma model. Although total MDSCs were elevated following SNAb treatment in the composite trauma model, closer analysis revealed that a subset of high side scatter MDSCs were depleted successfully. Single-cell RNA sequencing results suggest that the unexpected effects of SNAbs in the composite trauma model may be related to varied gene expression patterns of *S100a8/a9* in MDSC subsets. Elevated levels of systemic MDSCs and monocytes after SNAb treatment inversely correlated to healing while T cells, T helper cells, and cytotoxic T cells positively correlated, even just 48 h post-treatment. These correlations provide insight into why rats treated with SNAbs and BMP-2 may have demonstrated worse healing than rats treated with BMP-2 only. Altogether, these results provide further evidence in support of the role of systemic immune dysregulation and specifically MDSCs in impairing bone repair outcomes and suggest the need for further studies on these cells and their subsets to improve targeted immunomodulatory interventions and enhance healing.

## Data Availability

The original contributions presented in the study are included in the article/**Suplementary Material**, further inquiries can be directed to the corresponding author/s.

## References

[1] Zura R Xiong Z Einhorn T Watson JT Ostrum RF Prayson MJ Epidemiology of fracture nonunion in 18 human bones. JAMA Surg. (2016) 151(11):e162775. 10.1001/jamasurg.2016.2775 27603155

[2] Wildemann B Ignatius A Leung F Taitsman LA Smith RM Pesántez R Non-union bone fractures. Nat Rev Dis Prim. (2021) 7(1):1–21. 10.1038/s41572-020-00234-1 34354083

[3] Low EE Inkellis E Morshed S. Complications and revision amputation following trauma-related lower limb loss. Injury. (2017) 48(2):364–70. 10.1016/j.injury.2016.11.019 27890336

[4] Harris AM Althausen PL Kellam J Bosse MJ Castillo R. Complications following limb-threatening lower extremity trauma. J Orthop Trauma. (2009) 23(1):1–6. 10.1097/BOT.0b013e31818e43dd 19104297

[5] MacKenzie EJ Bosse MJ Pollak AN Webb LX Swiontkowski MF Kellam JF Long-term persistence of disability following severe lower-limb trauma: results of a seven-year follow-up. J Bone Jt Surg. (2005) 87(8):1801. 10.2106/JBJS.E.00032 16085622

[6] Caetano-Lopes J Canhão H Fonseca JE. Osteoimmunology - the hidden immune regulation of bone. Autoimmun Rev. (2009) 8(3):250–5. 10.1016/j.autrev.2008.07.038 18722561

[7] Rosenthal MD Moore FA. Persistent inflammation, immunosuppression, and catabolism: evolution of multiple organ dysfunction. Surg Infect (Larchmt). (2016) 17(2):167. 10.1089/sur.2015.184 26689501 PMC4790202

[8] Gentile LF Cuenca AG Efron PA Ang D. Persistent inflammation and immunosuppression: a common syndrome and new horizon for surgical intensive care. J Trauma Acute Care Surg. (2012) 72(6):1491–501. 10.1097/TA.0b013e318256e000 22695412 PMC3705923

[9] Huber-Lang M Lambris JD Ward PA. Innate immune responses to trauma. Nat Immunol. (2018) 19(4):327–41. 10.1038/s41590-018-0064-8 29507356 PMC6027646

[10] Binkowska AM Michalak G Slotwiński R. Current views on the mechanisms of immune responses to trauma and infection. Cent Eur J Immunol. (2015) 40(2):206–16. 10.5114/ceji.2015.52835 26557036 PMC4637396

[11] Kimura F Shimizu H Yoshidome H Ohtsuka M Miyazaki M. Immunosuppression following surgical and traumatic injury. Surg Today. (2010) 40(9):793–808. 10.1007/s00595-010-4323-z 20740341 PMC7101797

[12] Xiao W Mindrinos MN Seok J Cuschieri J Cuenca AG Gao H A genomic storm in critically injured humans. J Exp Med. (2011) 208(13):2581–90. 10.1084/jem.20111354 22110166 PMC3244029

[13] Vantucci CE Roy K Guldberg RE. Immunomodulatory strategies for immune dysregulation following severe musculoskeletal trauma. J Immunol Regen Med. (2018) 2:21–35. 10.1016/j.regen.2018.07.001

[14] Lai D Qin C Shu Q. Myeloid-derived suppressor cells in sepsis. Biomed Res Int. (2014) 2014:598654. 10.1155/2014/598654 24995313 PMC4065675

[15] Zhu X Pribis JP Rodriguez PC Morris SM Vodovotz Y Billiar TR The central role of arginine catabolism in T-cell dysfunction and increased susceptibility to infection after physical injury. Ann Surg. (2014) 259(1):171–8. 10.1097/SLA.0b013e31828611f8 23470573

[16] Condamine T Gabrilovich DI. Molecular mechanisms regulating myeloid-derived suppressor cell differentiation and function. Trends Immunol. (2011) 32(1):19–25. 10.1016/j.it.2010.10.002 21067974 PMC3053028

[17] Cheng A Krishnan L Pradhan P Weinstock LD Wood LB Roy K Impaired bone healing following treatment of established nonunion correlates with serum cytokine expression. J Orthop Res. (2018) 37(2):299–307. 10.1002/jor.24186 PMC7605215 30480339

[18] Cheng A Vantucci CE Krishnan L Ruehle MA Kotanchek T Wood LB Early systemic immune biomarkers predict bone regeneration after trauma. Proc Natl Acad Sci U S A. (2021) 118(8):e2017889118. 10.1073/pnas.2017889118 33597299 PMC7923361

[19] Vantucci CE Krishan L Cheng A Prather A Roy K Guldberg RE. BMP-2 delivery strategy modulates local bone regeneration and systemic immune responses to complex extremity trauma. Biomater Sci. (2021) 9(5):1668–82. 10.1039/D0BM01728K 33409509 PMC8256799

[20] Vantucci CE Ahn H Fulton T Schenker ML Pradhan P Wood LB Development of systemic immune dysregulation in a rat trauma model of biomaterial-associated infection. Biomaterials. (2021) 264:120405. 10.1016/j.biomaterials.2020.120405 33069135 PMC8117743

[21] Liu J Toy R Vantucci C Pradhan P Zhang Z Kuo KM Bifunctional janus particles as multivalent synthetic nanoparticle antibodies (SNAbs) for selective depletion of target cells. Nano Lett. (2021) 21(1):875–86. 10.1021/acs.nanolett.0c04833 33395313 PMC8176937

[22] Bah I Kumbhare A Nguyen L McCall CE Gazzar ME. IL-10 induces an immune repressor pathway in sepsis by promoting S100A9 nuclear localization and MDSC development. Cell Immunol. (2018) 332:32. 10.1016/j.cellimm.2018.07.003 30025864 PMC6129403

[23] Sinha P Okoro C Foell D Freeze HH Ostrand-Rosenberg S Srikrishna G. Proinflammatory S100 proteins regulate the accumulation of myeloid-derived suppressor cells. J Immunol. (2008) 181(7):4666–75. 10.4049/jimmunol.181.7.4666 18802069 PMC2810501

[24] Zhao F Hoechst B Duffy A Gamrekelashvili J Fioravanti S Manns MP S100a9 a new marker for monocytic human myeloid-derived suppressor cells. Immunology. (2012) 136(2):176–83. 10.1111/j.1365-2567.2012.03566.x 22304731 PMC3403264

[25] Cheng P Eksioglu EA Chen X Kandell W Le Trinh T Cen L S100A9-induced overexpression of PD-1/PD-L1 contributes to ineffective hematopoiesis in myelodysplastic syndromes. Leuk. (2019) 33(8):2034–46. 10.1038/s41375-019-0397-9 PMC6687540 30737486

[26] Silvin A Chapuis N Dunsmore G Goubet AG Dubuisson A Derosa L Elevated calprotectin and abnormal myeloid cell subsets discriminate severe from mild COVID-19. Cell. (2020) 182(6):1401–18.e18. 10.1016/j.cell.2020.08.002 32810439 PMC7405878

[27] Wang S Song R Wang Z Jing Z Wang S Ma J. S100a8/A9 in inflammation. Front Immunol. (2018) 9(JUN):1298. 10.3389/fimmu.2018.01298 29942307 PMC6004386

[28] Kolambkar YM Dupont KM Boerckel JD Huebsch N Mooney DJ Hutmacher DW An alginate-based hybrid system for growth factor delivery in the functional repair of large bone defects. Biomaterials. (2011) 32(1):65–74. 10.1016/j.biomaterials.2010.08.074 20864165 PMC3013370

[29] Bronte V Brandau S Chen SH Colombo MP Frey AB Greten TF Recommendations for myeloid-derived suppressor cell nomenclature and characterization standards. Nat Commun. (2016) 7:12150. 10.1038/ncomms12150 27381735 PMC4935811

[30] Sadtler K Estrellas K Allen BW Wolf MT Fan H Tam AJ Developing a pro-regenerative biomaterial scaffold microenvironment requires T helper 2 cells. Science (80-). (2016) 352(6283):366–70. 10.1126/science.aad9272 PMC4866509 27081073

[31] Spitzer MH Carmi Y Reticker-Flynn NE Kwek SS Madhireddy D Martins MM Systemic immunity is required for effective cancer immunotherapy. Cell. (2017) 168(3):487–502.e15. 10.1016/j.cell.2016.12.022 28111070 PMC5312823

[32] Raber P Ochoa AC Rodríguez PC. Metabolism of L-arginine by myeloid-derived suppressor cells in cancer: mechanisms of T cell suppression and therapeutic perspectives. Immunol Invest. (2012) 41(6–7):614–34. 10.3109/08820139.2012.680634 23017138 PMC3519282

[33] Elkabets M Ribeiro VSG Dinarello CA Ostrand-Rosenberg S Di Santo JP Apte RN IL-1β regulates a novel myeloid-derived suppressor cell subset that impairs NK cell development and function. Eur J Immunol. (2010) 40(12):3347–57. 10.1002/eji.201041037 21110318 PMC3373225

[34] Tu S Bhagat G Cui G Takaishi S Kurt-Jones EA Rickman B Overexpression of interleukin-1β induces gastric inflammation and cancer and mobilizes myeloid-derived suppressor cells in mice. Cancer Cell. (2008) 14(5):408–19. 10.1016/j.ccr.2008.10.011 18977329 PMC2586894

[35] Gazon H Barbeau B Mesnard JM Peloponese JM. Hijacking of the AP-1 signaling pathway during development of ATL. Front Microbiol. (2018) 8:2686. 10.3389/fmicb.2017.02686 29379481 PMC5775265

[36] Narumi K Miyakawa R Ueda R Hashimoto H Yamamoto Y Yoshida T Proinflammatory proteins S100A8/S100A9 activate NK cells via interaction with RAGE. J Immunol. (2015) 194(11):5539–48. 10.4049/jimmunol.1402301 25911757

[37] Wei F Zhou Y Wang J Liu C Xiao Y. The immunomodulatory role of BMP-2 on macrophages to accelerate osteogenesis. Tissue Eng - Part A. (2018) 24(7–8):584–94. 10.1089/ten.tea.2017.0232 28726579

[38] Lykissas M Gkiatas I. Use of recombinant human bone morphogenetic protein-2 in spine surgery. World J Orthop. (2017) 8(7):531–5. 10.5312/wjo.v8.i7.531 28808623 PMC5534401

[39] Conway JD Shabtai L Bauernschub A Specht SC. BMP-7 versus BMP-2 for the treatment of long bone nonunion. Orthopedics. (2014) 37(12):e1049–57. 10.3928/01477447-20141124-50 25437077

