# Supplementary figures and images for: Systemic Immune Modulation Alters Local Bone Regeneration in a Delayed Treatment Composite Model of Non-Union Extremity Trauma
Source: Front Surg. 2022 Jul 7;9:934773. doi: 10.3389/fsurg.2022.934773 (PMC9300902; doi:10.3389/fsurg.2022.934773)

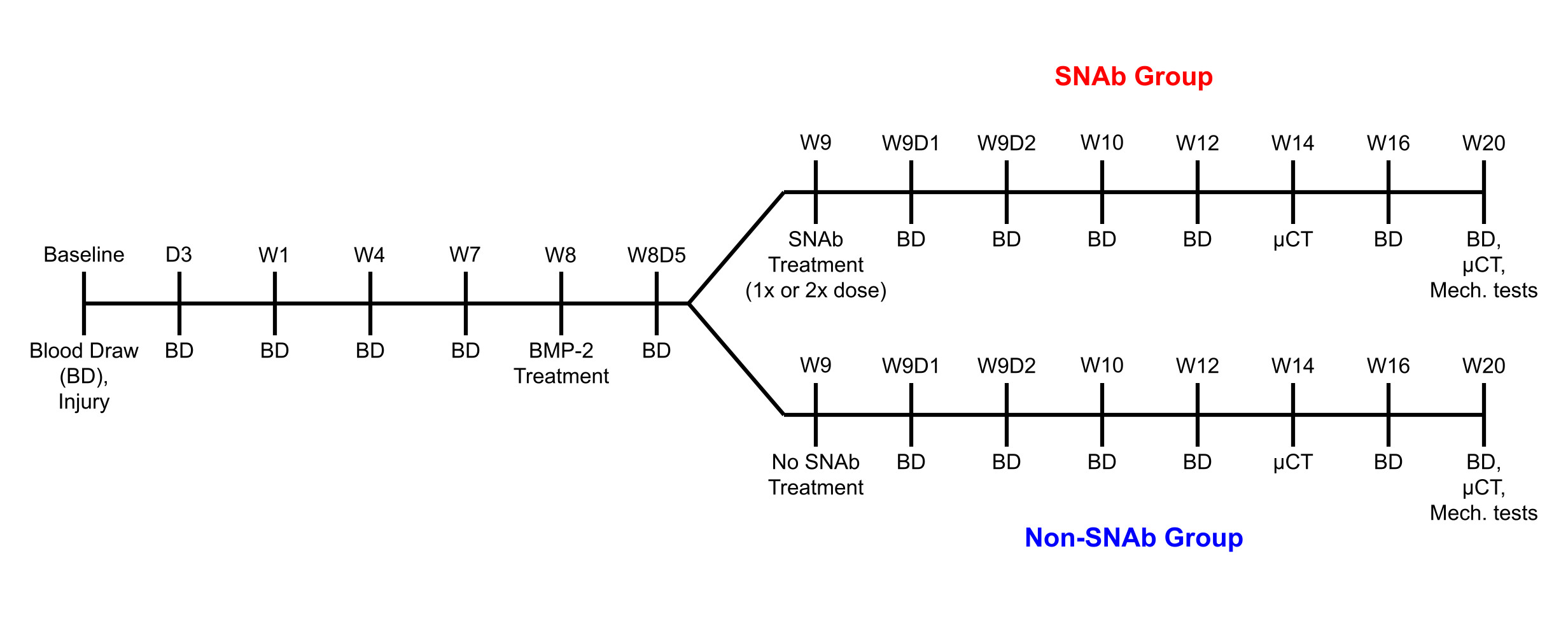

Supplement: Supplementary file 1 [file Image_1_v1.jpeg]

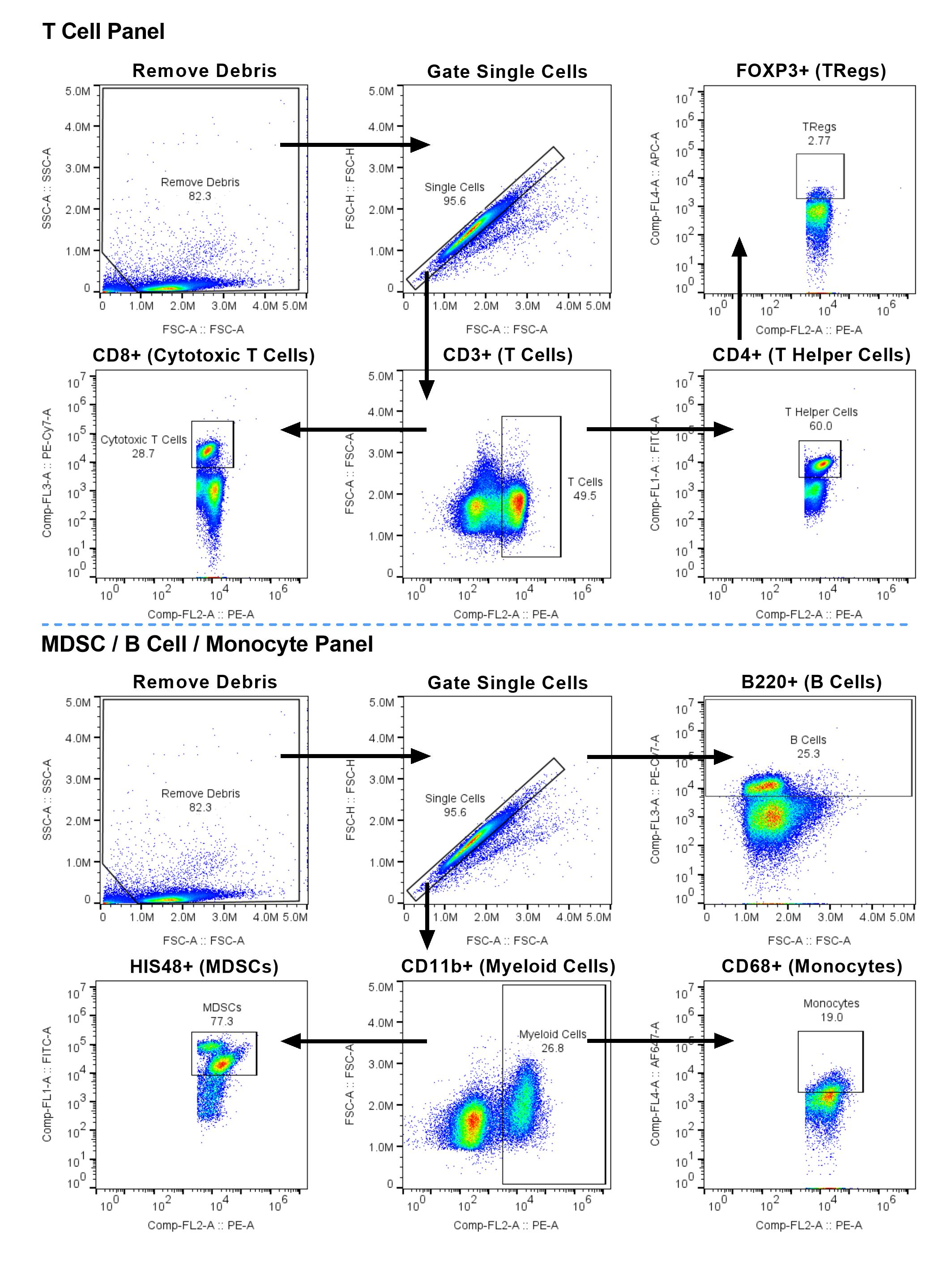

Supplement: Supplementary file 2 [file Image_2_v1.jpeg]

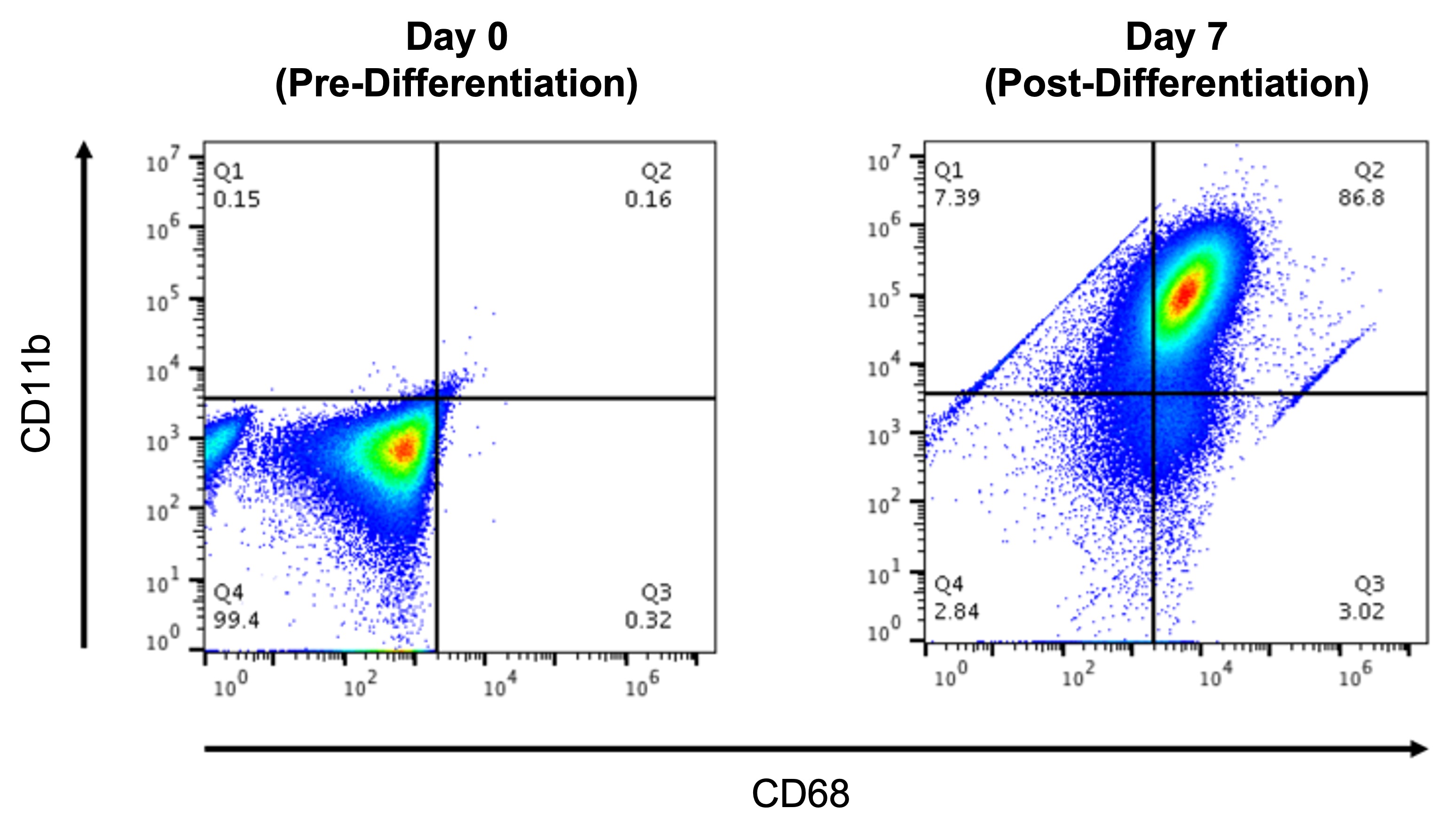

Supplement: Supplementary file 3 [file Image_3_v1.jpeg]

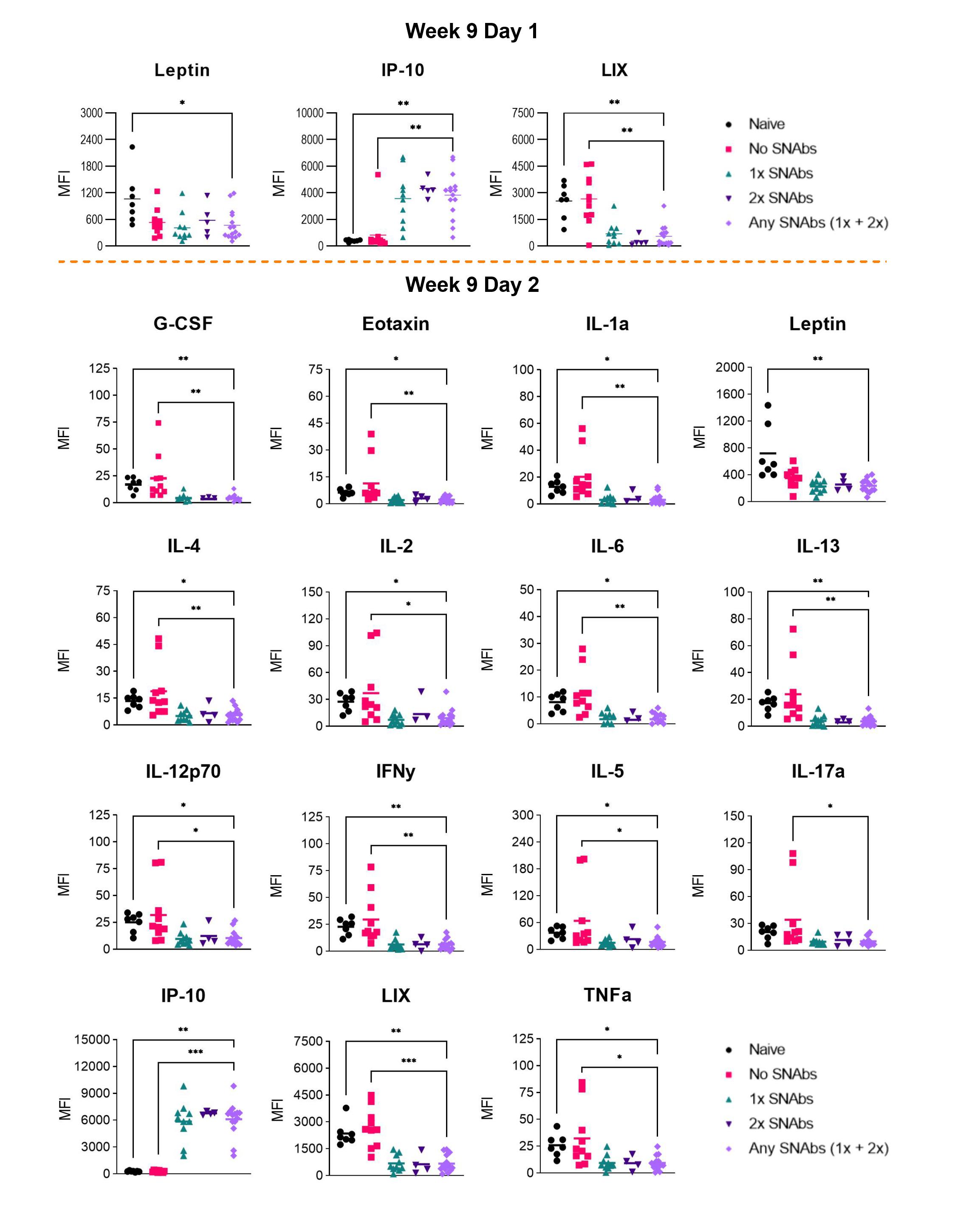

Supplement: Supplementary file 4 [file Image_4_v1.jpeg]

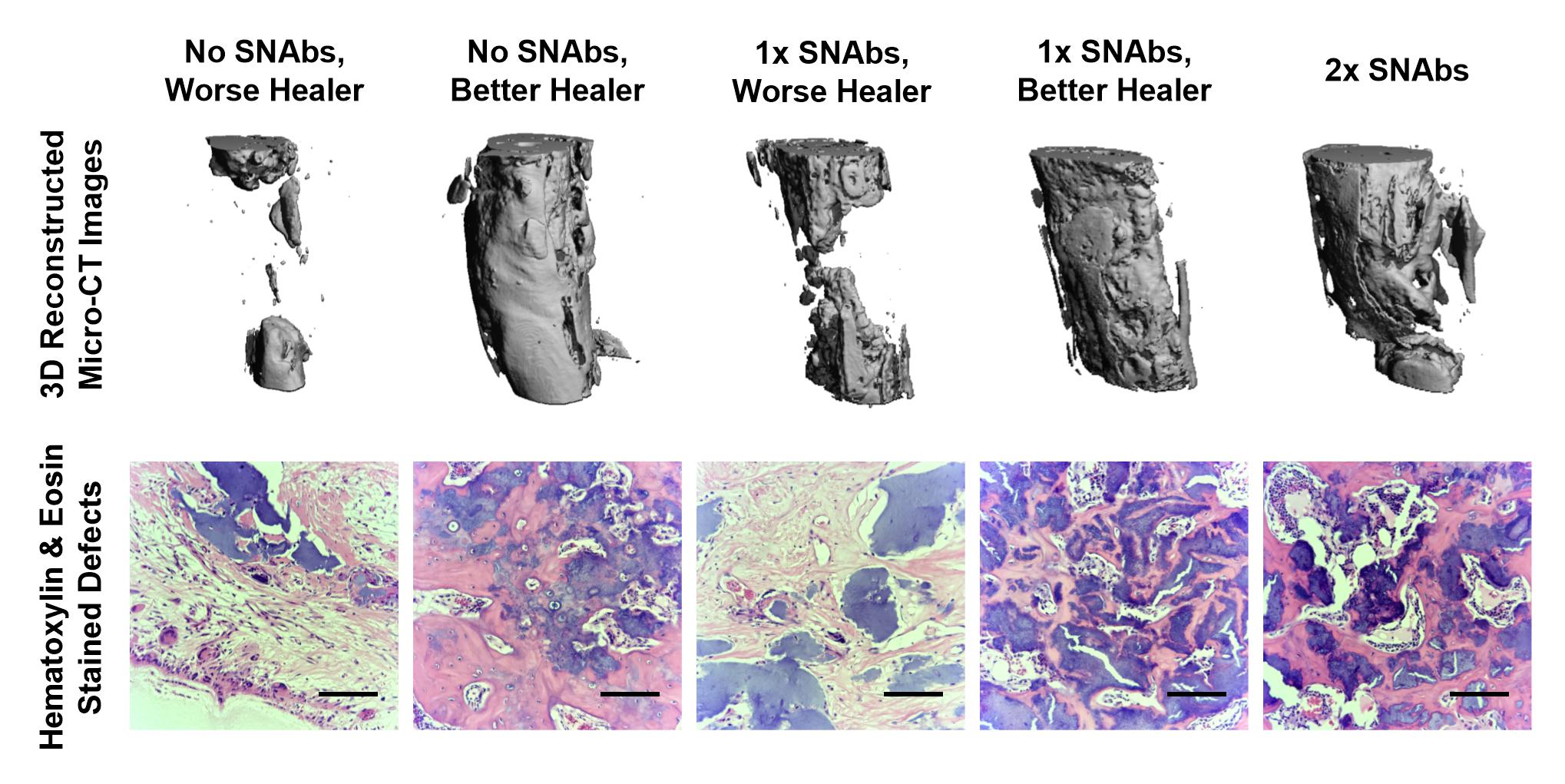

Supplement: Supplementary file 5 [file Image_5_v1.jpeg]

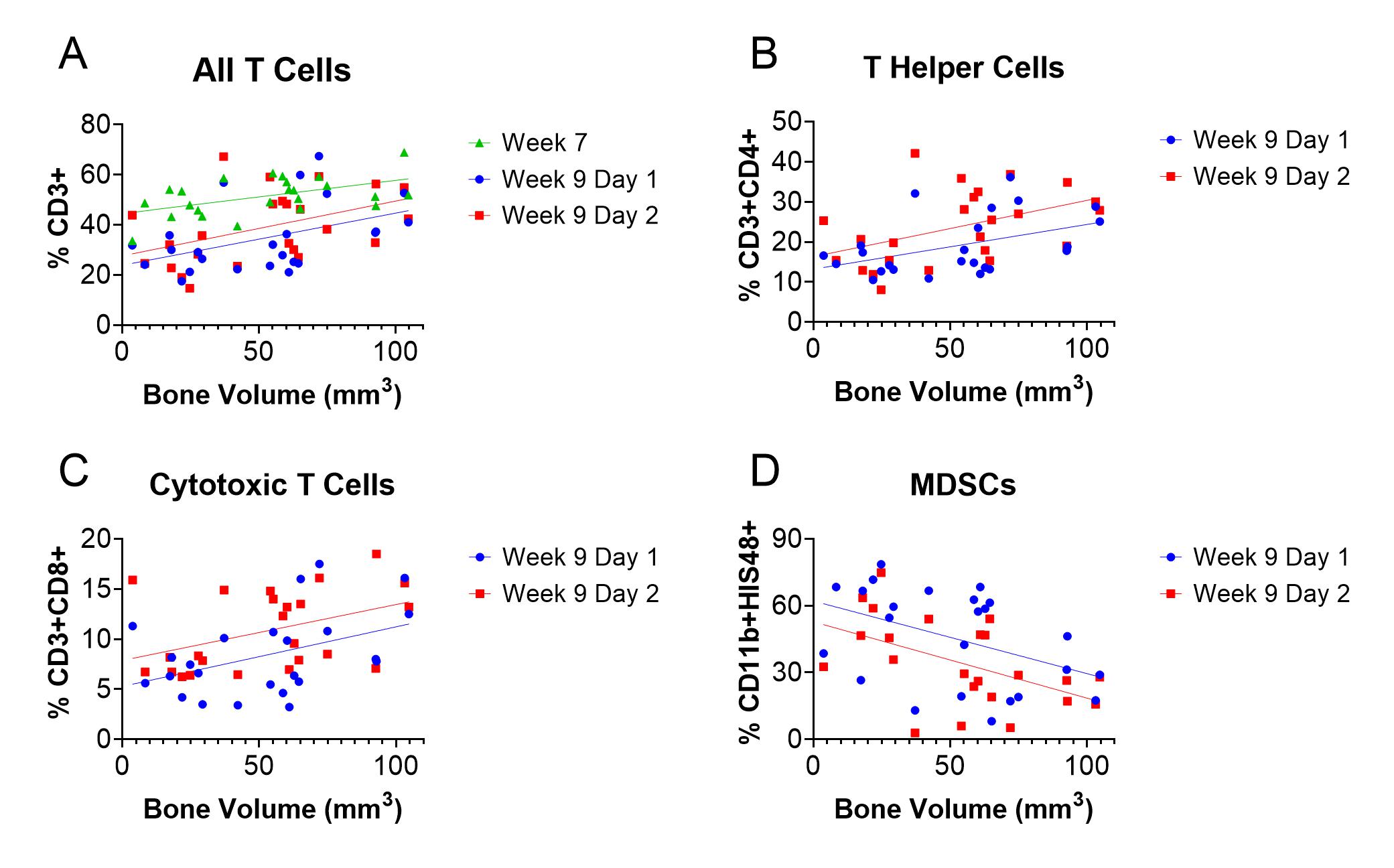

Supplement: Supplementary file 6 [file Image_6_v1.jpeg]
