# Supplementary material for: Systemic Immune Modulation Alters Local Bone Regeneration in a Delayed Treatment Composite Model of Non-Union Extremity Trauma
Source: Front Surg. 2022 Jul 7;9:934773. doi: 10.3389/fsurg.2022.934773 (PMC9300902; doi:10.3389/fsurg.2022.934773)
Supplement: Supplementary file 7 [file Table_1_v1.docx]

**Supplementary Table S1. List of antibodies used for flow cytometry.**

| **Target** | **Conjugate** | **Dilution** | **Supplier** | **Reference** |
| --- | --- | --- | --- | --- |
| CD32 | None | 1:25 | BD Biosciences | 550271 |
| CD3 | PE | 1:75 | Thermo Fisher Scientific | 12-0030-83 |
| CD4 | FITC | 1:75 | Thermo Fisher Scientific | 11-0040-82 |
| CD8a | PE-Cy7 | 1:75 | Thermo Fisher Scientific | 25-0084-82 |
| FOXP3 | APC | 1:75 | Thermo Fisher Scientific | 17-5773-82 |
| HIS48 | FITC | 1:75 | Thermo Fisher Scientific | 11-0570-82 |
| CD11b | PE | 1:75 | Thermo Fisher Scientific | 12-0110-82 |
| B220 | PE-Cy7 | 1:75 | Thermo Fisher Scientific | 25-0460-82 |
| CD68 | Alexa Fluor 647 | 1:75 | Bio-Rad Laboratories | MCA341A647 |
