# Supplementary material for: Systemic Immune Modulation Alters Local Bone Regeneration in a Delayed Treatment Composite Model of Non-Union Extremity Trauma
Source: Front Surg. 2022 Jul 7;9:934773. doi: 10.3389/fsurg.2022.934773 (PMC9300902; doi:10.3389/fsurg.2022.934773)
Supplement: Supplementary file 8 [file Table_2_v1.docx]

**Supplementary Table S2. Linear regression table describing the significant correlations shown in Figure 6.**

| Linear Regression | Blood  All T Cells  W9D2 | Blood  All T Cells  W20 | Blood  T Helper  W9D2 | Blood  T Helper  W20 | Blood  Cytotoxic T  W9D2 | Blood  Cytotoxic T  W20 | Blood  MDSCs  W9D2 | Blood  MDSCs  W20 | Blood  Monocytes  W9D2 |
| --- | --- | --- | --- | --- | --- | --- | --- | --- | --- |
| Slope | 0.4399 | 0.3846 | 0.2930 | 0.2518 | 0.1348 | 0.1230 | -0.5878 | -0.3061 | -0.07171 |
| Y-intercept | 9.973 | 29.91 | 4.224 | 19.06 | 2.923 | 10.36 | 76.25 | 31.84 | 10.01 |
| Pearson r | 0.7891 | 0.8573 | 0.7868 | 0.8733 | 0.8266 | 0.7292 | -0.7735 | -0.7503 | -0.8415 |
| P-value | 0.0199 | 0.0065 | 0.0205 | 0.0046 | 0.0114 | 0.0401 | 0.0244 | 0.0320 | 0.0176 |
